# Intratesticular leiomyoma: A case report and a literature review

**DOI:** 10.1016/j.ijscr.2020.05.030

**Published:** 2020-05-23

**Authors:** Skander Zouari, Mouna Ben Othmane, Khaireddine Bouassida, Wissem Hmida, Mehdi Jaidane

**Affiliations:** Urology Department, Sahloul Hospital, Sousse, Tunisia

**Keywords:** Leiomyoma, Testicle, Scrotal mass, Benign

## Abstract

**Introduction:**

Leiomyomas are benign tumors that originate from smooth muscles cells. Intratesticular leiomyoma is a very rare type of benign testicular masses. Through the literature, only 11 cases were reported.

**Presentation of case:**

A 36 years old man presented with painless right scrotal mass. Clinical examination showed right upper polar testicular mass, well circumscribed and highly vascularized on sonography. Serum tumor markers were normal. Patient was explained the possibility of an organ sparing surgery if frozen section shows no sign of malignancy. Midline incision with frozen section was performed, with wide local excision. Pathological examination of the specimen confirmed intratesticular leiomyoma. Two years after the surgery, patient showed no sign of recurrence.

**Discussion:**

Intratesticular Leiomyoma is usually revealed as a non-tender firm scrotal mass that increases in size. Sonography is the imaging modality of choice for assessing intrascrotal pathology, and cannot distinguish benign from malignant tumor. Surgical management is subject of debate. While some authors recommend radical orchiectomy, others suggest the use of frozen section intraoperatively, and perform conservative treatment by mass excision in the absence of signs of malignancy.

**Conclusion:**

Intratesticular leiomyoma is a very rare finding. When suspected, conservative management using frozen section followed by mass excision can be done, but final diagnosis remains on pathological examination and immunohistochemistry of the specimen.

## Highlights

• Testicular Leiomyomùa is a rare entity.
• Clinical presentation and physical examination are non specific, and cannot distinguish it from a testicular cancer.
• Imaging is based on ultrasound, which describes the features of the testicular leiomyoma precisely, but cannot make the diagnosis.
• Only pathological examination of the specimen after surgery can confirm the diagnosis.
• A frozen section of the mass followed by mass excision can lead to organ sparing surgery if the diagnosis is suspected intraoperatively.

## 1 Introduction

Most of the testicular masses are malignant. It is considered as malignant whenever a solid testicular mass is detected clinically or in sonography. However, some benign lesions exist as well such as benign teratoma, benign sex cord-stromal tumor (Leydig and Sertoli cell tumors), epidermoid cyst, and lipoma. Intratesticular leiomyoma is a very rare pattern of those benign lesions. So far, only 11 cases were reported in the literature. We herein report a case of intratesticular leiomyoma, with its sonographic, intraoperative and pathological features, and discuss both radical and conservative surgery, with a comprehensive literature review. The work has been reported in line with the SCARE criteria [1].

## 2 Case presentation

A 36 years old man presented with a painless right scrotal mass of 2 years duration without weight loss or other associated symptoms. Neither hematuria nor other lower urinary tract symptoms were reported by the patient. He had no past medical history. The mass continued to increase in size and volume becoming functionally uncomfortable. Therefore, he decided to see a urologist.

On physical examination, the palpation of the right testicle revealed a firm and painless upper polar mass of a diameter of 3 cm. The controlateral testicle was normal in size and in shape.

**Fig. 1:**
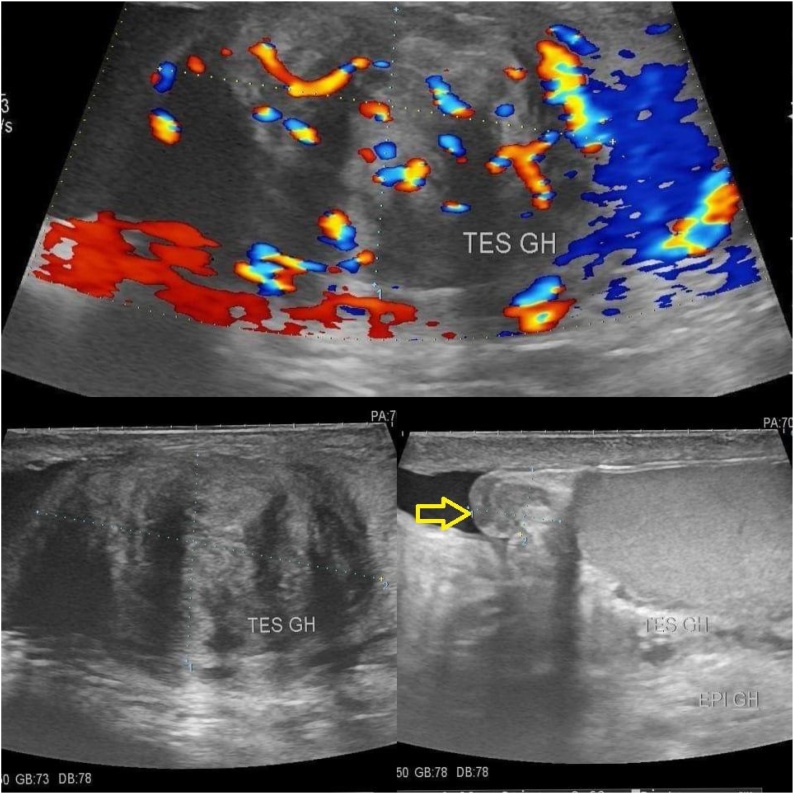
Ultrasound of the left testicle revealing a well circumscribed mass measuring 35 × 20 mm located in the upper pole.

Biologically speaking, the tumor markers (Lactate dehydrogenase, alpha fetoprotein and beta human chronic gonadotropin) were within the normal limits. The scrotal US showed well limited left mass, well circumscribed and with highly vascularized color doppler echostructure, measuring 35 × 20 mm (Fig. 1). The rest of the testicular parenchyma appears homogeneous. No varicocele or hydrocele was observed. Patient was explained the probability of malignant tumor to occur, and the possibility to make frozen section intraoperatively and then decide whether or not to remove the testicle. The patient agreed to the second option.

**Fig. 2:**
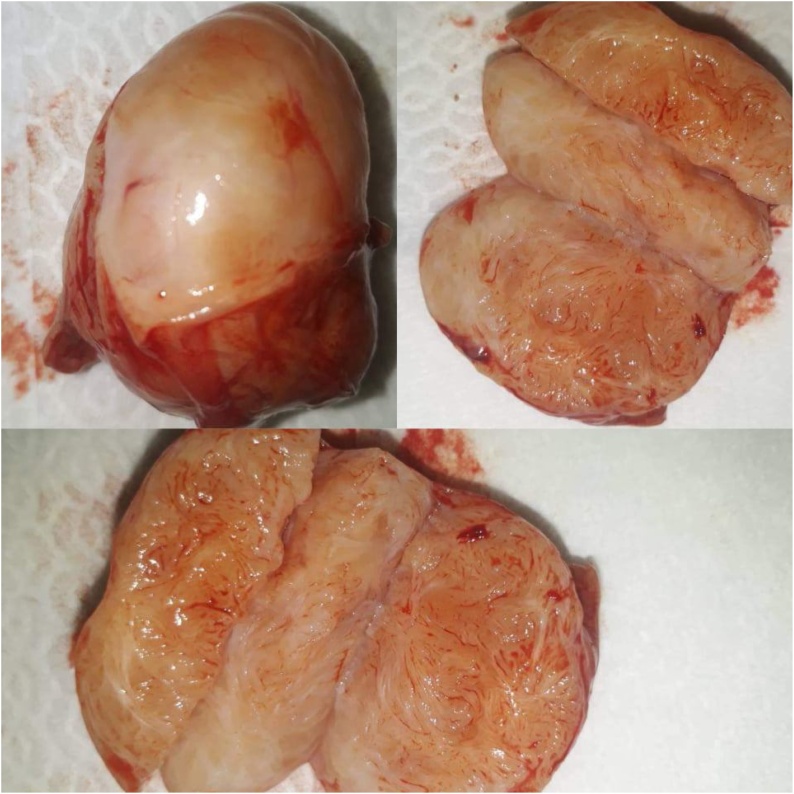
Intraoperative aspect of the specimen after mass excision. A well limited mass measuring 4 cms.

A midline incision over the median raphe was performed, and dissection of the scrotal layers was performed. An intratesticular mass of the upper pole was found, measuring approximatively 4 cm. A frozen section showed no sign of malignancy. Therefore, a wide local excision of the mass was performed with 1 cm of safety margin (Fig. 2). Post-operative course was uneventful and patient left the hospital the next day.

**Fig. 3:**
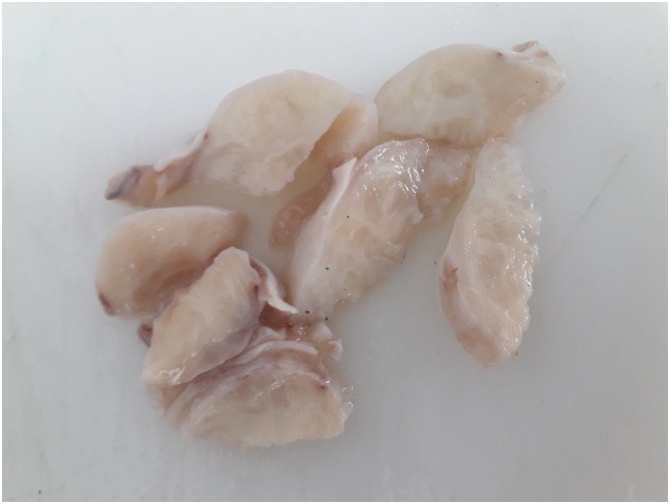
Gross examination of the tumor showing a nodular mass measuring 4 × 2 × 2cms, whitish in colour, with fasciculated aspect on section.

**Fig. 4:**
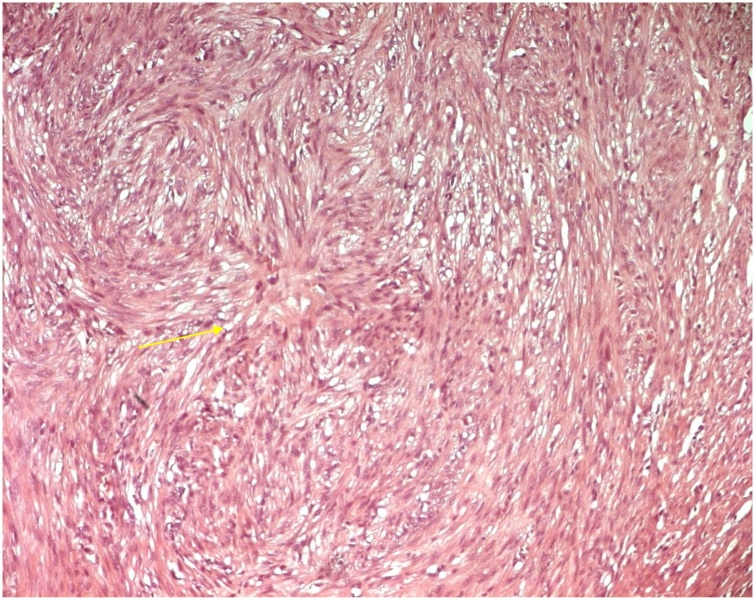
Microscopic examination showing a well limited mass, encapsulated, formed by smooth muscle tissue organized in interlaced bundles. The cells are elongated, with fusiform nuclei, with rounded ends, an inhomogenic chromatin, thin nucleoli and an eosinophilic cytoplasm with poorly defined cytoplasmic limits (Arrow).

Histologically speaking, gross examination revealed nodular mass measuring 4 × 2 × 2 cms, whitish in colour, with fasciculated aspect on section (Fig. 3). Microscopically, it showed well limited mass, encapsulated, formed by smooth muscle tissue organized in interlaced bundles. The cells are elongated, with fusiform nuclei, rounded ends, an inhomogenic chromatin, thin nucleoli and an eosinophilic cytoplasm with poorly defined cytoplasmic limits (Fig. 4). Cytonuclear atypias were exceptional and mitosis was absent. The final conclusion led to a 4 cms intratesticular leiomyoma without any sign of malignancy.

The patient was kept on both clinical and radiological follow up, and 2 years after the surgery, he showed no sign of local or distant recurrence.

## 3 Discussion

Leiomyomas are benign tumors that originate from smooth muscles cells. In the genitourinary system, they are most frequently located in the renal pelvis, but also can be seen in the bladder, spermatic cord, epididymis, prostate, glans penis or the scrotum [2]. The leiomyoma of the testis is extremely rare; the main hypothesis is that it arises from the contractile cells in the tunica propria of the seminiferous tubules [3]. Extensive literature review shows that leiomyoma of the tunica vaginalis, tunica albuginea, epididymis, spermatic cord, and body of the testis has been described [4]. Based on a systematic Pubmed search using the keywords “intratesticular leiomyoma”, 11 published cases were found in the literature. All the cases are summarized in (Table 1). The mean age of the patients was 45 years old, and it is usually revealed as a non-tender firm scrotal mass that increases in size like in our patient. It can sometimes be accompanied by hydrocele [3] but in our case, there were no associated symptoms other than functional discomfort. On examination, usually a painless firm mass developing in one of the poles of the testicle is found. The average size is 3 cm, ranging from 1 to 8. The tumor markers are generally normal, except for one case where slightly raised serum lactate dehydrogenase was found [5].

**Table 1:** Reported cases of intratesticular leiomyoma in the literature.

Author	Age	Side	Symptoms	Size	Serum Tumor Markers	Frozen Section	Treatment	Follow up (In Months)	Recurrence
Honore (1975)	65	Right	Firm non tender nodule	1 cm	Normal	No	Radical Orchidectomy	36	No
Nino Murcia (1989)	N/A	N/A	N/A	N/A	N/A	N/A	N/A	N/A	N/A
Takahashi (1991)	36	Right	Firm non tender nodule	3 cms	Normal	No	Radical Orchidectomy	N/A	N/A
Longchampt (1998)	N/A	N/A	N/A	N/A	N/A	N/A	N/A	N/A	N/A
Thomas et al. (1998)	52	Left	Painless increase of testicle size	6 × 4 × 3 cms	Normal	No	Radical Orchidectomy	N/A	N/A
Destito (1999)	N/A	N/A	N/A	N/A	N/A	No	Radical Orchidectomy	N/A	N/A
Gonzalez et al. (1999)	18	Right upper pole	Painless testicular Mass	1 cm	Normal	Yes	Mass Excision	12	No
O’brian (2008)	31	Left upper pole	Palpable testicular Mass	8 × 7 mm	Normal	Yes	Radical Orchidectomy	84	No
Kulloli et al. (2010)	40	Left lower pole	Painless Scrotal swelling	3 × 2 cms	Normal	No	Radical Orchidectomy	N/A	N/A
Yong et al. (2015)	47	Left upper pole	Non tender scrotal swelling	1 × 1 cm	LDH raised AlphaFP and bHCG Normal	No	Mass excision	N/A	N/A
Baker (2019)	74	Right Pole	Mild Scrotal discomfort	0.5 × 0.5 × 0.6 cms	Normal	No	Radical Orchidectomy	No	No
**Present Case (2019)**	**36**	**Right pole**	**Painless scrotal swelling**	**4 cms**	**Normal**	**Yes**	**Mass excision**	**24**	**No**

N/A: not available.

Keyword search: intratesticular leiomyoma.

Sonography is the imaging modality of choice for assessing intrascrotal pathology, and the case of leiomyoma; it is described as an hypoechoic intratesticular mass, well limited. But as other benign lesions except for the epidermoid cyst of the testis, it cannot be diagnosed by ultrasonography or magnetic resonance imaging, as it has the same sonographic aspect as malignant tumors, which represent the majority of testicular tumors [6].

Therefore, the final diagnosis is confirmed after microscopic examination of the specimen. For most of the authors, radical orchidectomy is performed through inguinal incision, based on the fact that benign lesion cannot be distinguished clinically from the more common testicular malignancy [4]. For Heidenreich and al, this should not be the standard approach if benign lesion is suspected, and microscopic examination of a frozen section can be made intraoperatively [7]. Hass and al. demonstrated that approximatively 20% of all radical orchiectomies performed for suspected disease did not meet the final expected pathology [8]. Two studies evaluated the accuracy of frozen section in the correct diagnosis of a testicular mass and showed excellent results [9,10]. Through the literature review, Gonzales and al [11]. chose inguinal incision while Yong and al [5]. performed midline incision over the raphe median as in our case. The first option is more suitable since it respects oncological principles.

Concerning pathological features, intratesticular leiomyoma is described with the presence of elongated spindle shaped cells with eosinophilic cytoplasm. The nuclei are thin and oval in shape and are usually seen at the center of the cell. There is an edematous stroma interspersed with rich capillary vascular supply. The cells tend to be packed and overlapping and can be arranged in intertwining fasciculi. In immunohistochemistery, it stains positively for desmin and smooth muscle actin [12]. The prognosis is excellent, and no case of recurrence has been reported so far.

## 4 Conclusion

Intratesticular leiomyoma is a very rare finding. The diagnosis is made on pathological examination, and because it is impossible to distinguish it clinically from malignant tumors, most authors stand for radical orchidectomy as the treatment of choice. Perhaps a more conservative approach for benign intratesticular masses can be successfully achieved under certain prerequisites, as frozen section in this case.

## Declaration of Competing Interest

The authors have no conflict of interest to declare.

## Funding

This research did not receive any specific grant from funding agencies in the public, commercial, or not-for-profit sectors.

## Ethical approval

Given the nature of the article, a case report, no ethical approval was required.

## Consent

Written informed consent was obtained from the patient for publication of this case and accompanying images. A copy of the written consent is available for review by the Editor-in-Chief of this journal on request.

## Author contribution

Skander Zouari: Writing - original draft.

Mouna Ben Othmane: Writing - review & editing.

Khaireddine Bouassida: Project administration.

Wissem Hmida: Supervision and reviewing.

Mehdi Jaidane : Supervision; Reviewing and editing.

## Registration of research studies

This does not apply as it is a case report of a patient who has given written consent and has been de-identified. It is therefore not prospective research involving human participant.

## Guarantor

Dr. Skander Zouari.

## Provenance and peer review

Editorially reviewed, not externally peer-reviewed.
